# ^68^Ga and ^188^Re Starch-Based Microparticles as Theranostic Tool for the Hepatocellular Carcinoma: Radiolabeling and Preliminary *In Vivo* Rat Studies

**DOI:** 10.1371/journal.pone.0164626

**Published:** 2016-10-14

**Authors:** Elise Verger, Pierre Drion, Geneviève Meffre, Claire Bernard, Luc Duwez, Nicolas Lepareur, Olivier Couturier, François Hindré, Roland Hustinx, Franck Lacoeuille

**Affiliations:** 1 INSERM UMR-S 1066 MINT (Micro- et Nano-médecines Biomimétiques), University of Angers, Angers, France; 2 Nuclear Medicine department, CHU de Liège, University of Liège, Liège, Belgium; 3 Animal Facility, Experimental Surgery, GIGA-R & Credec, University of Liège, Liège, Belgium; 4 Nuclear Medicine Department, Centre de Lutte Contre le Cancer (CLCC) Eugène Marquis, INSERM U991, Rennes, France; 5 Nuclear Medicine department, CHU d'Angers, University of Angers, Angers, France; 6 PRIMEX (Plateforme de Radiobiologie et d'IMagerie EXperimentale), University of Angers, Angers, France; Helsingin Yliopisto, FINLAND

## Abstract

**Purpose:**

This work aims to develop, validate and optimize the radiolabeling of Starch-Based Microparticles (SBMP) by ^188^Re and ^68^Ga in the form of ready-to-use radiolabeling kits, the ultimate goal being to obtain a unique theranostic vector for the treatment of Hepatocellular Carcinoma.

**Methods:**

Optimal labeling conditions and composition of freeze-dried kits were defined by monitoring the radiochemical purity while varying several parameters. *In vitro* stability studies were carried out, as well as an *in vivo* biodistribution as a preliminary approach with the intra-arterial injection of ^68^Ga radiolabeled SBMP into the hepatic artery of DENA-induced rats followed by PET/CT imaging.

**Results:**

Kits were optimized for ^188^Re and ^68^Ga with high and stable radiochemical purity (>95% and >98% respectively). The *in vivo* preliminary study was successful with more than 95% of activity found in the liver and mostly in the tumorous part.

**Conclusion:**

SBMP are a promising theranostic agent for the Selective Internal Radiation Therapy of Hepatocellular carcinoma.

## Introduction

Hepatocellular Carcinoma (HCC), a liver cancer is the fifth most common cancer and the second leading cause of cancer death in men worldwide (seventh and sixth for women respectively) [1]. HCC is associated with a poor prognosis, with a 5-year survival of 12% [2]. Depending on the stage of the disease and other prognostic parameters [3–6] several therapeutic options are available but the only curative treatment is surgery, either resection or transplant [2,7–9].

Selective Internal Radiation Therapy (SIRT) is a relatively new therapeutic modality [10] that is increasingly applied in both primary and secondary liver tumors [11]. This technique consists of the injection of yttrium-90 (^90^Y) microspheres to the tumor through the hepatic arteries that vascularize the tumor in order to deliver high radiation dose while sparring the healthy hepatic parenchyma that is supplied by the venous system [12–15]. ^90^Y is a pure β^-^ emitter (E_max_ = 2.3 MeV) with a half-life of 64h (2.67 days) [16] and a short tissue penetration (mean 2.5 mm and maximum 11mm) [17]. SIRT is a two-step procedure, with a pre-therapeutic step carried out before the therapy. A pre-treatment assessment is first performed for evaluating the tumor vasculature, the potential extra-hepatic uptake and the hepatopulmonary shunt. Following a diagnostic hepatic arteriography, a catheter is positioned selectively according to tumor localization and therapeutic objectives. Then, human serum albumin macroaggregates (MAA) labeled with ^99m^Tc are injected via this catheter in order to mimic the biodistribution of the ^90^Y-microspheres that will be injected for therapy in the second step of the procedure [18]. Scans post infusion of ^99m^Tc-MAA allow firstly, the exclusion of patients with a high percentage of radioactivity being shunted into the lungs or spread into any other organ, such as the gastroduodenum and secondly, the determination of the predictive tumor dosimetry. Both steps (pre-therapeutic and therapy itself) are based on the assumption that the biodistribution of ^90^Y-microspheres and ^99m^Tc-MAA will be identical. However, the MAA have a different size and morphology (aggregates with heterogeneous shapes with size comprised between 10–100μm [19]) in comparison with the ^90^Y-microspheres (spherical shape with size comprised between 20–40μm [20]) that can lead to a different biodistribution and an approximate tumor dosimetry [11,15,18,21,22].

In order to overcome this problem we propose an original theranostic tool, the starch-based microparticles (SBMP), as a unique system for both the pre-therapeutic step after technetium-99m (^99m^Tc) or gallium-68 (^68^Ga) radiolabeling and the treatment after rhenium-188 (^188^Re) radiolabeling. These radionuclides offer the advantage of being all easily obtained from generators (^99^Mo/^99m^Tc; ^68^Ge/^68^Ga and ^188^W/^188^Re) and do not require an on-site cyclotron like fluor-18 (^18^F) [23–26]. ^99m^Tc is a pure gamma emitter (E_max_ = 140 keV; t_½_ = 6.02h) while ^68^Ga is a β^+^ emitter (E_max_ = 1.9 MeV; t_½_ = 68min); they are used for diagnostic purpose to perform Single Photon Emission Computed Tomography (SPECT) and Positron Emission Tomography (PET) imaging respectively. ^188^Re is a β^-^ emitter (E_max_ = 2.1MeV; t_½_ = 16.98h) allowing the radiotherapy but emits also a γ emission (155 keV) that can be imaged. SBMP was first developed and patented in our laboratory for lung perfusion scintigraphy and formulated as ready-to-use ^99m^Tc radiolabeling kit [27–29].

These microparticles display several attractive features as theranostic vector for the SIRT of HCC, i.e. a non-human/non-animal origin that ensures a safety toward the risk of disease transmission and an ability for complexation of radioisotopes. Using the same particle in both steps of the SIRT constitutes a major advantage, as the biodistribution of the diagnostic and therapeutic agents should be identical. Furthermore the SBMP labeled with ^68^Ga for the imaging and dosimetry with PET offer improved sensitivity, spatial resolution and signal quantitation compared with SPECT [30–32]. Finally, the SBMP labeled with ^188^Re for the treatment allow a very easy post-treatment imaging in order to verify the distribution of the therapeutic agent. Hence, this leads us to consider the SBMP as a promising theranostic vector for the SIRT of HCC.

In this work we were able to develop and optimize ready-to-use kits allowing fast and stable labeling of SBMP with ^188^Re or ^68^Ga. For the diagnostic kit, we labeled the already optimized ^99m^Tc-SBMP kit with ^68^Ga, and subsequently developed a dedicated kit for ^68^Ga. An *in vivo* biodistribution by PET/CT (PET/Computed Tomography) of the SBMP radiolabeled with ^68^Ga injected intra-arterially in a carcinogen-induced HCC rat model was also carried out as a very preliminary test of the pre-therapeutic step, prior to the mandatory comprehensive biodistribution and dosimetry studies whose completion is required before considering human use.

## Materials and Methods

### Materials

Potato starch was a kind gift from Roquette Freres (Lestrem, France). Sodium metaperiodate, sodium borohydride, stannous chloride dihydrate (SnCl_2_, 2H_2_O), sodium gluconate and cadaverine were purchased from Sigma Aldrich (USA). Medical argon gas was obtained from Air Liquide Santé (Gentilly, France), and deionized water was delivered by a Milli-Q plus system (Merck Millipore, Darmstadt, Germany). Solution of sodium perrhenate (^188^ReO_4_^-^) was obtained by saline elution of a ^188^W/^188^Re generator (IRE, Belgium) and then concentrated. Solution of gallium trichloride (^68^GaCl_3_) is obtained from a ^68^Ge/^68^Ga generator (50mCi IGG-100, Eckert&Ziegler, Germany), based on the TiO_2_ resin technology. It is eluted with 5mL of 0.1N HCl solution obtained from HCl 30% suprapure (Merck Millipore, Germany) diluted in ultrapure water (J.T.Baker, USA). Hydrophilic 5 mm syringe filters were purchased from Sartorius (Palaiseau, France). Sodium acetate trihydrate 1M (pH = 4.1) was provided by Eckert&Ziegler (Germany).

### Starch-Based MicroParticles (SBMP) synthesis

Microparticles were prepared according to a previously described and patented method [29]. Briefly, native starch microparticles of the desired size were selected by sieving, then oxidized by sodium periodate to obtain dialdehyde starch. This step allows the grafting of a polyamine ligand the cadaverine onto the modified starch particles. This grafting is stabilized by a reduction step with sodium borohydride to obtain the SBMP. Amino functions from the cadaverine act as the complexant agent for radiometals like ^99m^Tc, ^188^Re and ^68^Ga.

#### Size measurement

Size distribution measurements of the particles were carried out through a counting analyzer Multisizer 3 Coulter Counter (Beckman Coulter, France). Five to 10 mg of SBMP were suspended in a weak Isoton II electrolyte solution (Beckman Coulter, France) and drawn through a small aperture (200 μm) until at least 20,000 particles were counted.

### Formulation of starch-based microparticles as ready-to-use kits

Ready-to-use kits were prepared by adding the desired amount of SBMP, 1mL of NaCl 0.9% and if needed amount of SnCl_2_ and gluconate to a sterile vial according to the different experiments carried out. The microparticles were then lyophilized and kept under vacuum.

### Radiochemical purity measurements

Radiochemical purity was assessed according to a previously described method [27]. It consists of a filtration of 0.5mL to 1mL of the labeled microparticles suspension on a 5-μm syringe filter. The RCP is determined with the following equation:

$$RCP\;(\%)=\frac{Filter\;activity\;(MBq)}{Filter\;activity\;(MBq)+Filtrate\;activity\;(MBq)}\times 100$$

### ^188^Re radiolabeling

#### Determination of the optimal composition of ^188^Re labeling kits

In order to determine the influence of each parameter on the RCP different variations of the composition of kits and labeling conditions have been tested. The SnCl_2_ is a reducing agent required during the radiolabeling process of the SBMP with ^99m^Tc [27,28] as well as ^188^Re [16,33–35]. To define the optimal amount needed for the complexation of the ^188^Re with the SBMP, a range of SnCl_2_ from 50μg to 2mg (*n* = 3) has been tested on kits containing an amount of 300mg SBMP. Then, the appropriate quantity of particles was assessed with kits containing 1mg of SnCl_2_ and a variation from 50 to 400mg of SBMP (*n* = 3). Influence of the amount of radiolabeling activity was tested with 100MBq and 3GBq and kits of 300mg SBMP with 1mg of SnCl_2_ (*n* = 3). A final optimization step was carried out with the addition of a range of sodium gluconate from 0 to 60mg (*n* = 3) in the selected 300mg SBMP kits. The gluconate is a weak chelate that allows the formation of a transitional complex and facilitate the complexation of ^188^Re with the ligand [36]. Apart from the variations, in most of the experiments SBMP kits have been labeled with about 100MBq of perrhenate, 2mL or 4mL of reaction volume and 30min of vortex agitation (Vortex-Genie 2T, Scientific Industries, USA) before the measure of RCP.

#### *In vitro* Stability study

Kits containing 300mg of SBMP, 1mg of SnCl_2_ and 9mg of NaCl (*n* = 3) were labeled with 2.5mL of perrhenate (3107 ± 422 MBq) followed by a vortex agitation of 30min. After 1h of radiolabeling the first RCP is measured followed by the measures at 6h and 24h, in order to determine the stability of the radiolabeling over time. After the first RCP, 2 aliquots of 0.5mL were removed to be incubated either with 4mL of rat plasma during 1h at 37°C or with 4mL of histidine 10^-3^M a metallic ion chelator during 3h at room temperature.

## ^68^Ga radiolabeling

### Direct labeling with ^68^GaCl_3_ eluate

Native starch particles labeling: 20mg of native starch particles (*n* = 3) were labeled with gallium trichloride (GaCl_3_) (552 ± 223 MBq) with addition of NaCl or HCl 0.1N if needed to obtain a 4mL reaction volume and put under vortex agitation. RCP was taken at different time (5, 10 and 30 min) by filtration.

SBMP labeling: 20mg (*n = 6*), 50mg (*n = 3*) or 100mg (*n = 3*) of SBMP and kits (originally optimized for ^99m^Tc radiolabeling [27,28]) containing 100mg of SBMP; 9mg of NaCl and 55μg of SnCl_2_ were labeled with 4mL of ^68^GaCl_3_ (552±120MBq and 785±171MBq for the 100mg kits) and put under vortex agitation. RCP was taken at different time (5, 10 and 30min).

#### Optimization of labeling with ^68^Ga with addition of pH4.1 buffer

Kits of 20mg of SBMP and 9mg of NaCl; 50mg of SBMP and kits of 100mg of SBMP and 9mg of NaCl were labeled with 4mL of ^68^GaCl_3_ (522±36MBq) and 0.5mL of sodium acetate trihydrate 1M (pH4.1) and put under vortex agitation (*n = 3*). RCP was taken at 5, 10 and 30min. The pH was measured on RCP filtrate with pH-indicator strips MColorpHast 2.0–9.0 (Merck Millipore, Germany).

#### *In vitro* stability studies

Kits were labeled with 4.5mL of ^68^GaCl_3_ (698±33MBq) under vortex agitation. After the first measure of the RCP at 5min, 0.5mL of Sodium Acetate trihydrate 1M was added to the kit to increase the pH from 1 up to 4 and obtain an injectable solution. The RCP was monitored at different times (5, 10, 30 (*n* = 3) and 60min (*n = 2*)).

Histidine Challenge: Just after the addition of sodium acetate to the kits, 1mL of SBMP (i.e. 20mg of microparticles) was incubated with 4mL of histidine (10^-3^M) (Sigma-Aldrich, USA) at room temperature under vortex agitation during 30min (*n* = 3). Labeling stability was assessed by measuring the RCP both before the incubation and during the incubation with histidine at 5, 10 and 30min.

Fetal Bovine Serum (FBS) stability: Likewise, just after the addition of Sodium acetate in the kits, 1mL of SBMP (i.e. 20mg of microparticles) was incubated with 4mL of FBS at room temperature under vortex agitation during 30min (*n* = 3). FBS is a complex media that contained among other proteins the transferrin, a natural iron chelator that can also chelate the ion Ga^3+^. The RCP was monitored before and after the incubation with FBS (5, 10 and 30min).

### *In vivo* studies

#### Ethic Statement

All experimental procedures and protocols used in this investigation were reviewed and approved by the Institutional Animal Care and Use Ethics Committee of the University of Liège (Belgium). The “Guide for the Care and Use of Laboratory Animals”, prepared by the Institute of Laboratory Animal Resources, National Research Council, and published by the National Academy Press, was followed carefully as well as European and local legislation. Animal welfare was assessed at least once per day, and all efforts were made to minimize animal suffering during the experiments (mainly housing conditions as no suffering appeared).

#### HCC induction model

Hepatic carcinogenesis was chemically induced in seven-weeks-old male Wistar rats weighting 180-296g obtained from the central animal facility of Liège University Hospital (Breeder agreement LA2610359). Diethylnitrosamine (DENA) (Sigma-Aldrich, USA) was administered in drinking water (100mg/L) during 8 weeks [37]. The animals were kept in polycarbonate cages containing a disposable polyethylene plastic liner (Tecniplast, Italy) with 2 animals per cage in a room with controlled temperature (20–22°C), humidity (50–70%), and light (12-hour light/dark cycles). Room air was renewed at the rate of 10 vol/hour. Tap water and food were provided *ad libitum*.

#### ^68^Ga radiolabeling of SBMP kits

Ready-to-use kits containing 20mg of SBMP, 55μg of SnCl_2_ and 9mg of NaCl were radiolabeled with 4mL of ^68^GaCl_3_ (626±55MBq) under vortex agitation. After adding 0.5mL of sodium acetate trihydrate 1M and checking the RCP by filtration the syringe was filled with the radiolabeled SBMP solution when the RCP has reached at least 98%. For 2 rats, 100 or 50μL of methylene blue was added in the syringe to facilitate the following of the radiolabeled solution during injection.

#### Intra-arterial injection of SBMP labeled with ^68^Ga

The totality of the procedure was realized under general anesthesia until sacrifice. Three rats were successfully injected 10±1 weeks after the end of the induction. Rats were weighted and sequentially anesthetized by i.p. injection of sodium pentobarbital (60 mg/kg of body weight) (Nembutal sodium solution, Abbott Laboratories, North Chicago, IL) followed by isoflurane anesthesia (1.5% isoflurane, 3% oxygen, Abbott Laboratories, USA) until euthanasia. The surgical procedure was performed under aseptic conditions and under a dissecting microscope. A laparotomy was performed to expose the liver and the hepatic arteries. The celiac trunk, hepatic and gastroduodenal arteries were identified and all the visible collateral arteries of the hepatic artery ligatured in order to prevent the particles to spread in other organs than the liver. The injection of the ^68^Ga-SBMP (21±26MBq) was realized directly in the celiac trunk by using a 27G ½ 0.4x13 needle (BD, USA) connected to a 1mL syringe with the aim that the solution reached the hepatic artery and the liver. At the moment of perforation the artery was clamped to temporarily stop arterial flow. The flow was restored during the injection, then after the injection a final ligature of the celiac trunk was performed. The rat muscular wall and skin were stitched up before the PET/CT imaging. After final imaging the rat were kept under anesthesia and euthanatized by a lethal injection of sodium pentobarbital (200 mg/kg of body weight) (Nembutal sodium solution, Abbott Laboratories, North Chicago, IL). The liver was harvested and preserved in formalin 4% (Q-Path Chemicals, VWR Chemicals, USA).

#### PET/CT imaging

The *in vivo* biodistribution of the ^68^Ga-SBMP was monitored with a clinical Gemini TF16 PET/CT scanner (Philips Medical Systems, The Netherlands). Static image acquisitions were performed 17min±1 after injection of the ^68^Ga-SBMP. Rats were maintained under isoflurane anesthesia (1.5% isoflurane, 3% oxygen, Abbott Laboratories, USA) during the acquisition time of 20min. The method of reconstruction used had the TOF (time-of-flight) capability disabled because of the small size of the animals [38]. Images were analyzed using PMOD (version 3.607). Volumes-of-interests (VOI) for liver, for non-specific activity in the intraperitoneal area and for the whole body were set manually with help of isovolumetric tool.

### Statistical Analysis

Statistical analyses were conducted using Prism 6.0 software (v6.0f, GraphPad software, La Jolla, USA). When testing the null hypothesis, a threshold value for p of 0.05 (error α) was chosen to set statistical difference. Anova test followed by a Tukey’s HSD test was used to perform multiple comparisons of the RCP means for rhenium-188 and gallium-68 radiolabeling studies.

## Results

### ^188^Re radiolabeling of SBMP

To develop the optimal ^188^Re radiolabeling kit, variation in several parameters were studied and the RCP was monitored. Thus varied amount of SnCl_2_ needed for the kit was tested along with the particle quantity, activity and gluconate addition. The SBMP have a mean size of 29.65±11.73μm and 1g of SBMP contains 87.64.10^6^ particles.

**Fig 1:**
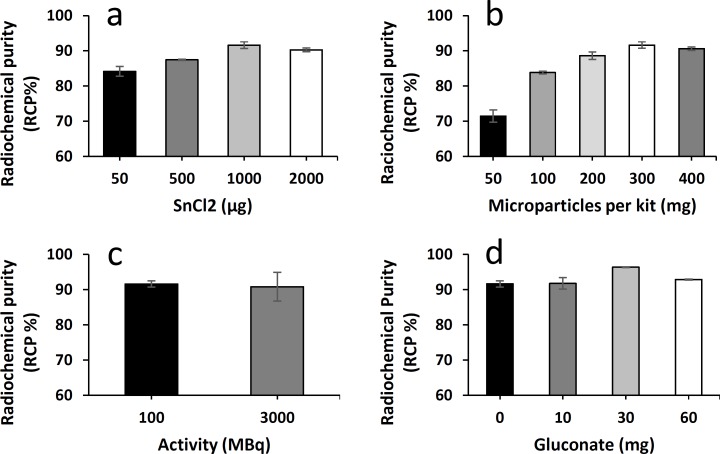
Influence on the RCP of several parameters during the ^188^Re radiolabeling of SBMP. (**a**) RCP of 300mg SBMP radiolabeled kits with varied amount of SnCl_2_. (**b**) RCP of 1mg SnCl_2_ radiolabeled kits with varied amount of SBMP. (**c**) RCP of 300mg SBMP, 1mg SnCl_2_ kits with varied amount of activity. (**d**) RCP of 300mg SBMP, 1mg SnCl_2_ kits with varied amount of gluconate.

A range of SnCl_2_ quantity from 50μg to 2000μg, was tested on 300mg SBMP kits to define the best reducing conditions for the complexation of ^188^Re (Fig 1A). The maximal RCP (91.6±0.9%) was obtained with kits containing 1000μg of SnCl_2_ and was significantly higher to RCP obtained with 50μg of SnCl_2_ (p<0.0001) and with 500μg of SnCl_2_ (p<0.0018). Adding a greater amount of SnCl_2_ (2000μg) did not cause a significant change in radiochemical purity (p = 0.2839). For further studies the amount of SnCl_2_ per kit was therefore set to 1000μg. The influence of particles quantity on RCP was assessed with increasing amount of SBMP from 50mg to 400mg (Fig 1B). The RCP increases with the quantity of SBMP up to 300mg. The maximal RCP (91.6±0.9%) was obtained with kits containing 300mg of SBMP and was significantly higher to RCP obtained with 50mg of SBMP (p<0.0001), 100mg of SBMP (p<0.0001) and 200mg of SBMP (p<0.0321). Adding a higher amount of SBMP (400mg) did not cause significant change in radiochemical purity (p = 0.742).

This composition of 300mg of SBMP and 1mg of SnCl_2_ was chosen to conduct the further investigations. The ability of the SBMP kit to complex high activity of ^188^Re was tested (Fig 1C). The RCP were not significantly different (p = 0.4602) whether the radiolabeling studies were conducted with high (3097±345MBq) or low (116±16MBq) activities.

Kits containing 300mg of SBMP; 1mg of SnCl_2_ were labeled with 3GBq of perrhenate and underwent stability study over time and competition tests in rat plasma and histidine 10^-3^M a metallic ion chelator (Table 1). The radiolabeling is stable over time and in competition.

**Table 1:** Stability over time and competitions tests of 300mg SBMP kits with 1mg of SnCl_2_ radiolabeled by ^188^Re.

	Stability over time			Competition test	
	Time after radiolabeling (h)			Incubation time	
	1	6	24	Rat plasma +1h	Histidine +3h
**RCP (%)**	88.8±0.5	88.0±0.7	83.8±5.4	84.4±1.5	83.7±1.3

In order to optimize radiolabeling of the SBMP kit, the influence of addition of gluconate to the kit was tested (Fig 1D). The RCP reached 96.4±0.1% with the addition of 30mg of gluconate and was significantly superior to the addition to the SBMP kit of 10mg of gluconate (p = 0.0013), 60mg of gluconate (p = 0.0071) or no gluconate (p = 0.0011).

### ^68^Ga radiolabeling of SBMP

**Fig 2:**
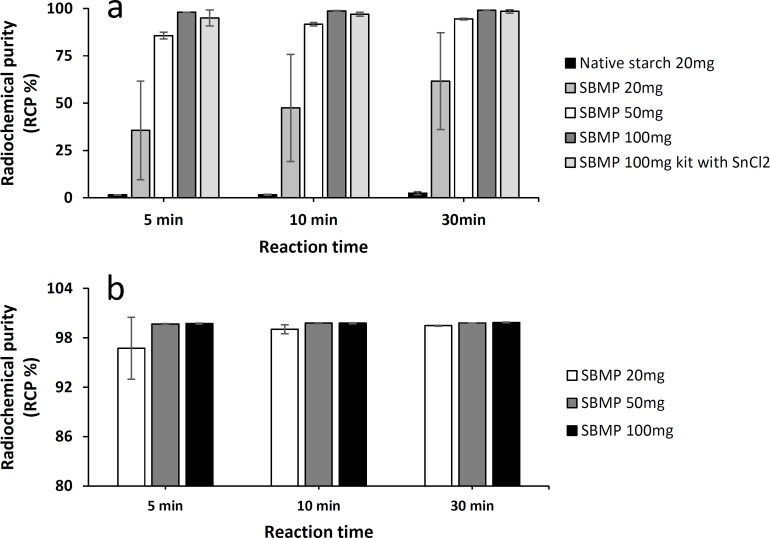
^68^Ga radiolabeling of SBMP. (**a**) Direct radiolabeling of varied amount of SBMP or native starch with ^68^GaCl_3_ eluate. (**b**) Optimization of ^68^Ga radiolabeling of varied amount of SBMP with addition of 0.5mL of sodium acetate trihydrate 1M (pH4.1).

The study of complexation of ^68^Ga by microparticles was first carried out by using directly a ^68^GaCl_3_ eluate without any buffer at room temperature (Fig 2A). The kinetic over time of the radiolabeling of ^68^Ga was monitored with the measure of the RCP 5min, 10min and 30min after the beginning of the radiolabeling reaction. As shown on Fig 2A native starch particles does not react with ^68^Ga since the RCP does not reach 3%, whereas SBMP were successfully radiolabeled. Using direct labeling from ^68^GaCl_3_ eluate the quantity of SBMP had an influence on the RCP. Indeed the RCP kinetic of 20mg of SBMP radiolabeled with ^68^GaCl_3_ was slow and the maximal RCP did not reach 70% giving a RCP between 35.6±26.1% and 61.6±25.6% (Fig 2A). The radiolabeling improved when using a kit containing 50mg of microparticles, with a RCP = 91.7±1.0% after 10min of reaction, and reached a RCP = 94.5±0.3% after increasing the time of reaction to 30 min (p = 0.0218). However an even better kinetics of labeling reaction was obtained with kits containing 100mg of SBMP as the RCP reached 98.7±0.2% after 10min of reaction (p = 0.0001) and was maximal (99.1±0.1%) after 30min (p = 0.0002). We observed a similar kinetic with 100mg SBMP kits already optimized for the ^99m^Tc containing 55μg of SnCl_2_, the reducing agent needed for ^99m^Tc or ^188^Re [16,33–35].

The optimized radiolabeling of SBMP by ^68^Ga was achieved by adding sodium acetate trihydrate 1M (pH = 4.1) in the kit just after the addition of ^68^Ga eluate (Fig 2B). These labeling conditions allowed a high and stable kinetic of radiolabeling with RCP>95% from 5min and ≥99% from 10min for whatever was the amount of microparticles in the kit with no statistical difference between 20mg, 50mg and 100mg. For the same amount of sodium acetate trihydrate (0.5mL), we observed slight variations in the pH according to the quantity of SBMP with a pH ranging from 3.0–3.6 for 20mg; 3.9 for 50mg and a suitable pH for injection of 4.0 with 100mg of SBMP.

*In vitro* stability studies (Table 2) showed a stable radiolabeling with a high RCP of almost 100% both in a complex media such as Fetal Bovine Serum and also in histidine solution a metallic ion chelator. This study mimics the stability in blood after injection. As control the RCP was monitored in parallel with the unchallenged SBMP up to 60min and the RCP was also >99%.

**Table 2:** *In vitro* stability studies: ^68^Ga radiolabeling of 100mg SBMP kit with 55μg of SnCl_2_.

	Radiochemical purity			
***Radiolabeling time***	5 min	10 min	30 min	60min
**Stability over time**	95.6±2.2%	99.7±0.1%	99.9±0.1%	99.9% (100%; 99.8%)
***Incubation time***	5min	10min	30min	
**Histidine competition**	99.6±0.2%	99.8±0.1%	99.8±0.1%	
**FBS competition**	99.3±0.2%	99.5±0.1%	99.2±0.1%	

### *In vivo* studies of the ^68^Ga-SBMP

**Fig 3:**
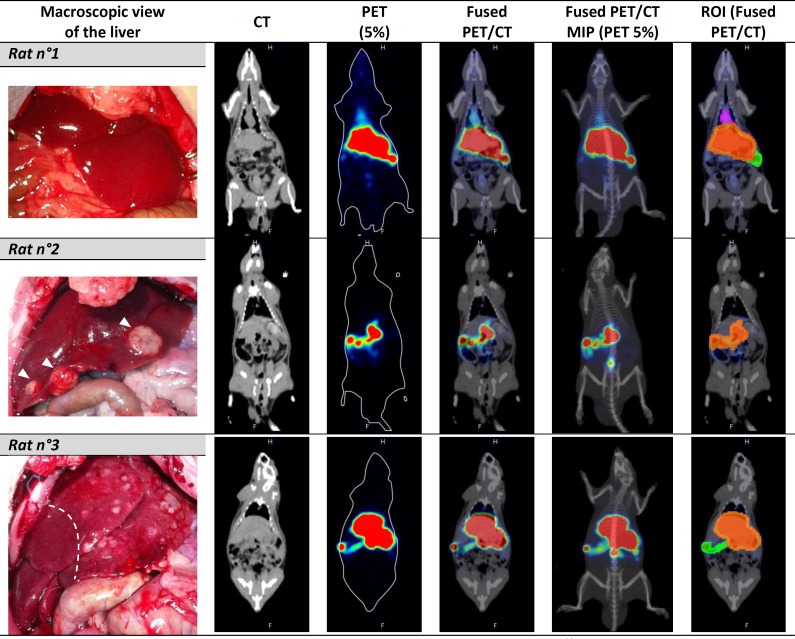
PET/CT imaging of DENA-induced rats injected intra-arterially with ^68^Ga-SBMP and macroscopic view of the liver. On the macroscopic view, (open abdominal view) the major tumors nodules are identified by white *arrowhead*s for the rat n°3 and for the rat n°8 the *dashed line* delimits the upper tumorous part of the liver. In the column displaying the ROI on the corresponding PET/CT slice: the *orange ROI* corresponds to the liver, the *green* one to the intraperitoneal area and the *pink* one to the activity counted as the rest of the body. *Abbreviations*: *CT (Computed Tomography); PET (Positron Emission Tomography); MIP (Maximum Intensity Projection) and ROI (Region of Interest)*.

A bioevaluation of the ^68^Ga-SBMP was carried out in 3 rats induced with a carcinogen to develop HCC tumor, as a preliminary study of the SIRT’s pre-therapeutic diagnostic step, in order to evaluate the behavior, stability and biodistribution of the particles *in vivo* (Fig 3). For this experiment, kits of 20mg of SBMP (optimized for ^99m^Tc labeling i.e. with 55μg of SnCl_2_) were labeled with 626MBq±55MBq with addition of 0.5mL of sodium acetate trihydrate 1M (pH = 4.1) with a RCP>98%. The PET/CT imaging shows that all injections resulted in the particles being locally gathered in the liver with no spreading throughout the body (Fig 3). This is confirmed by the PET biodistribution analysis: the ^68^Ga-SBMP are concentrated in the liver with a mean of 96.4±0.9% (rat n°1: 97.4%; n°2: 96.3% and n°3: 95.6%) of the total activity in the rat, with the rest of the activity in the intraperitoneal area surrounding the liver with a mean of 3.4±1.2% (rat n°1: 2.1%; n°2: 3.7% and n°3: 4.4%). No activity was found in the rest of the body (mean of 0.2±0.3% with rat n°1: 0.5% and the 2 others rats: 0.0%). Previous studies have shown that the intravenous injection of the SBMP results in a biodistribution in the lungs with all of the microparticles trapped in the lungs capillaries [27]. Since no activity in the lungs was observed, this supports a stability of the SBMP and the fact that they stay confined to the liver microvasculature with no recirculation. The macroscopic aspect of the liver showed that among the 3 rats, one of them did not present any lesions (rat n°1) despite the DENA induction. The second rat (n°2) had well defined tumor nodules rather large with several smaller lesions across the whole liver with a surrounding parenchyma with a smooth aspect. The rat n°3 displayed a widely spread HCC in the left lobes of the liver with a granular aspect while the right lobe was not affected by tumor. The radiolabeled particles in the rat without any lesion (n°1) were distributed homogeneously throughout the whole liver whereas the activity in the two other rats was found mostly in the tumorous parts of the liver (Fig 3).

## Discussion

In the context of HCC, the development of a unique radiolabeled vector for both the pre-therapeutic evaluation (^68^Ga-SBMP) and the treatment itself (^188^Re-SBMP), could solve the issues of miscorrelation in the biodistribution encountered in the clinical practice when using ^99m^Tc-MAA and the ^90^Y-microspheres [11,18,21,22].

The size of SBMP vary from 7 to 60μm with a mean of 30μm [27,28]. The average size is comparable to the ^90^Y-micropsheres currently used in SIRT. However the size distribution of the SBMP is different, with some smaller particles, which theoretically could lead to a distribution further downstream in the liver microvasculature. This may favorably modify the microscopic dosimetry but this aspect will require appropriate comparative studies with ^90^Y-microparticles. By their non-human and non-animal origins they avoid risk of disease transmission. The range of tissue penetration and E_max_ is similar between ^90^Y and ^188^Re. The ^90^Y (E_max_ = 2.3 MeV) have a maximum range of 12mm and a mean of 2.8mm while the ^188^Re (E_max_ = 2.1 MeV) have a maximum range of 11mm and a mean of 2.4mm [39,16]. ^188^Re half-life is shorter than ^90^Y (17.0 h versus 64.1 h respectively). However, contrary to ^90^Y, a pure beta emitter, the ^188^Re has the advantage to possess a gamma emission with a similar energy than ^99m^Tc (E_max_[^99m^Tc] = 140keV and E_max_[^188^Re] = 155keV, 15% [16,39]) allowing a SPECT imaging of the particles after the injection in an easiest way than with ^90^Y. Post-treatment imaging is important in order to evaluate the accurate delivery of the therapeutic dose: verify the distribution of the particles, the absence of extrahepatic deposition and predict the treatment efficacy [20,40–42]. ^90^Y can be indirectly imaged by SPECT through bremsstrahlung radiations that are created when the β particles interact with the matter, but with poor spatial resolution and result only in approximate evaluation. A gain in resolution can be gained with PET by imaging the very small amount of positron (0.003%) emitted through the decay of ^90^Y [43–47]. The direct imaging of ^188^Re could offer an easier and better quantitative assessment.

Our previous study has demonstrated that SBMP was not sensible to the enzymatic degradation by the alpha-amylase [28]. In the lungs the half-life of SBMP approached 3h due to mechanical degradation with the forces of friction encountered in the lungs. It is an advantage for patients in lung perfusion scintigraphy, since the time for the acquisition is already sufficient. These forces of friction are not present in the liver and we did not observe any SBMP degradation *in vitro* or *in vivo* during our investigations. However the *in vivo* stability of the compounds is of primary importance, especially considering the therapeutic one, i.e. the SBMP radiolabeled with ^188^Re, and further investigation in animal models is needed.

The optimized ^188^Re dedicated kits (300mg of SBMP, 1mg of SnCl_2_, 9mg of NaCl and 30mg of gluconate) contain a higher quantity of SnCl_2_ than ^99m^Tc dedicated kits (1mg versus 55μg); this is due to the fact that perrhenate is more difficult to reduce than perrtechnetate [16,33]. The addition of 30mg of gluconate in the kits seems to help the reaction of complexation. The gluconate is a weak chelate that can form transitional complex with the perrhenate and reduce its oxidation state to facilitate the complexation [36].

The SBMP kit offers advantages and flexibility. The ^188^Re is easily available from in-house generator and cost-effective [48–50]. The production of the ^90^Y-microspheres depends directly of a cyclotron and currently their very high cost is a challenge for a broader use [39,48]. Moreover the ^188^Re labeling process of SBMP is fast, performed at room temperature and the activity per particles can be varied and adjusted according to the amount of activity put into the kit. The ^188^Re kit of 300mg contains approximately 26 million of particles. With a RCP of 96.4% and perrhenate elution activities between 3GBq and 22GBq [48], theoretical specific activity of SBMP may range from 114Bq/particle to 811Bq/particle. This places the SBMP between the SIR-Sphere® (50Bq/particle) and the TheraSphere® (2500Bq/particle) [17,18,20,51]. They offer an intermediate alternative to overcome the limitations encountered in some cases with the two clinical devices. The lower specific activity of SIR-Sphere® imply a higher number of particles injected (approximately 40–80 million microspheres per treatment) and this could lead to the saturation of the entire vascular bed with an incomplete administration of the therapeutic dose [51–53] and a potential limitation of effect of the radiations due to hypoxia. Indeed, oxygenated cells are more sensible to the radiations than hypoxic cells [54,55]. On the contrary, TheraSphere® have a high specific activity per microsphere, hence a lower number of particles needed for treatment (approximately 2–4 million microspheres) [51]. This prevent the problems of saturation and the oxygenation is maintained [56] but an inadequate coverage of the tumor volume could occur [51–53]. The aim of an ideal device would be to obtain an optimal balance between the number of particles (the embolic effect) and the therapeutic dose injected (the radioactive effect) [53] to achieve a good biodistribution (coverage of all the tumor volume by the particles) without reaching a saturated state of the entire vascular bed that would prevent the full administration of the intended dose [51,57]. The characteristics of the ^188^Re-SBMP kit constitute a promising alternative for the SIRT of HCC.

SBMP kits for the ^99m^Tc radiolabeling have already been optimized for lung perfusion scintigraphy but our results prove that they are also very appropriate for the radiolabeling with ^68^Ga. Furthermore the ^68^Ga radiolabeling of the SBMP is an improvement in the development of an optimized radiotheranostic vector for SIRT. In comparison to the clinically used ^99m^Tc-MAA, the ^68^Ga-SBMP provides two advantages: (i) the vector is the same for the pre-therapeutic step and the therapy (SBMP), and (ii) ^68^Ga allows the use of PET which is more accurate for dosimetry than SPECT, with better sensitivity and spatial resolution [30–32]. Hence, the use of ^68^Ga-SBMP would provide a more precise and quantitative assessment of the biodistribution, thus allowing for improved individual dosimetry and therapy planning. The use of ^99m^Tc-SBMP instead of ^68^Ga-SBMP is also possible for the pre-therapeutic step if no PET is available, but the ^68^Ga-SBMP should be preferred when both SPECT and PET are available.

The fact that native starch are not labeled by ^68^Ga highlights the importance of the ligand grafted onto the SBMP for the radiometals’ chelation. The ligand, a polyamine, allows the complexation of both the ^188^Re (most likely in its +5 oxidation form) and the ^68^Ga (in +3 oxidation state) by the numerous nitrogen atoms that create good conditions as hard donors for chelation [58,16,59]. The labeling of SBMP by ^68^Ga with a RCP≥95% or higher was achieved with several labeling conditions, at room temperature and within 5 min of reaction. In the case of direct radiolabeling the higher and stable RCP≥98% were obtained with 100mg of SBMP only, corresponding to 8.8 million of particles. The direct radiolabeling of 100mg kits dedicated to ^99m^Tc containing 55μg of SnCl_2_ was also successful with a slightly slower kinetic that can be due to the reducing agent. The optimized labeling conditions were attained with the addition of sodium acetate trihydrate 1M, a pH = 4.1 buffer. Not only has it allowed to optimize the ^68^Ga radiolabeling of all SBMP quantities tested but with the increased pH of the radiolabeled solution, it is thereby injectable. The ligand grafted onto the SBMP, the cadaverine is very basic, hence variations in pH were observed with the same volume of acetate added. With a greater amount of SBMP the amount of acetate required to reach pH that is acceptable for injection is lower, as shown with the 100mg SBMP kits. With acetate condition even the 20mg kits could (without or with SnCl_2_ in the case of the *in vivo* study) attain a RCP>95% and even higher, whereas it only reached 60% of RCP after 30min with direct labeling. Again the kinetic is faster and reaches higher RCP without SnCl_2_. During our investigations we have observed that the best way to obtain the higher RCP was to add the acetate just after the ^68^GaCl_3_ and not before. The *in vitro* stability studies consistently showed a very stable labeling, as we never observed any decrease in the RCP after the maximum value is reached. During our investigation we did not see any influence of the amount of activity on the RCP as seen with perrhenate. The activity tested ranged from 367 up to 930MBq that correspond to the range obtained through the life of the ^68^Ge/^68^Ga generator.

The *in vivo* preliminary study was carried out in rats induced with DENA, a chemical carcinogen to develop HCC, this model offer the advantage of being histopathologically similar to human [60–62]. Thus the HCC nodules would be irrigated by arteries whereas the healthy parenchyma is irrigated by the venous system. However, the disadvantage of this model is its small size, particularly for interventional endovascular procedures. This first *in vivo* test was performed as a very preliminary evaluation of the biodistribution and stability of the SBMP after the intra-arterial injection. The ^68^Ga-SBMP were used for this first preliminary study in rats, as it would first be used in the pre-therapeutic step of SIRT for diagnostic, dosimetry and therapy planning purpose. In this context, we did not use the ^188^Re since as a therapeutic radionuclide it would leads to radioprotection constraints too demanding for an initial preclinical evaluation. Moreover it should be noted that the intra-arterial injection in the rat model is a major technical challenge in itself. In place of the catheterization of the femoral artery via angiographic monitoring [15], a surgical intervention was carried out with ligature of the collateral arteries except the hepatic artery. The direct injection of the radiolabeled particles into the celiac trunk was difficult. One problem encountered when injecting the particles was the tendency of SBMP to easily sediment on the syringe by gravity. Like the ^90^Y-microspheres used clinically [42,51] they are microparticles in suspension and like every microparticles easily sediment in time without agitation. To perform the injection of the ^90^Y-microspheres, dedicated infusion-sets for TheraSphere® and SIR-Sphere® have been conceived. These sets allow a slow perfusion matching the hepatic arterial flow rate [51] with a right suspension of the particles preventing sedimentation. However in our case, the difficulty is that no agitation can be done during the injection if the particles sediment again. The clinical infusion-sets are not appropriate to be used with a rat model. Thus injected activity and quantity of particles varied from one rat to another. A maximal activity of 51MBq was successfully injected in the last rat (n°3) after a continuous agitation of the particles in the syringe just before injection. For all the injections there was no spreading of the radiolabeled solution outside the artery except for the rat n°2 for which a part of the solution has extravasated to the abdominal cavity due to a slight damage of the celiac trunk during the injection. The extravasated solution being colored could be mostly removed. All injections resulted in the ^68^Ga-SBMP being locally gathered in the liver with more than 95% of the total activity. Moreover in the rats which developed the HCC, the activity was particularly found in the tumorous parts of the liver. This can be explained by the use of intra-arterial injection and the fact that liver tumors are irrigated mostly by the arteries whereas healthy parenchyma is irrigated by the venous system [12–15]. In the rat (n°1) no change in the blood supply occurred since no carcinogenesis developed [63]. This explains the homogeneous distribution of the particles observed in the liver. Moreover when injected intravenously, SBMP are trapped in the lungs capillaries [27]. No activity was found in the lungs, supporting the fact that SBMP do not recirculate. The activity observed in the rest of the body is negligible in the rat n°1 (0.5%) and is not observed in the other rats. The limited intraperitoneal activity may be due to thin non-ligatured collateral arteries that were not recognized during the surgery. The outcome of the injection is dependent of the success of the ligature of all the small collateral arteries. In some cases the ligature of all the collateral is difficult or even impossible. In any event, the results support the preliminary study that the particles are deposited in the liver since no activity was found that could be explained by blood distribution beyond the liver arterial circulation.

As observed previously, due to the difficulty of the intra-arterial injection in a small size model such as the rat, further studies need to be conducted in a bigger model, more appropriate to the interventional procedures. Hence, in perspective, in order to lead this work toward the clinic, further preclinical biodistribution and efficacy studies will be carried out in the rabbit with the VX2 model [64]. The injection would be easier in the femoral artery with the use of angiography.

## Conclusion

This work shows that the SBMP is a promising new theranostic agent for the SIRT of HCC. They can be labeled by 3 different radionuclides, ^99m^Tc [27,28], ^68^Ga and ^188^Re, at room temperature with fast labeling, with the potential of being used with SPECT and PET as well as for therapeutic purpose.

## Abbreviations

CT: Computed Tomography
DENA: Diethylnitrosamine
FBS: Fetal Bovine Serum
HCC: Hepatocellular Carcinoma
MAA: Albumin Macroaggregates
MIP: Maximum Intensity Projection
PET: Positron Emission Tomography
RCP: Radiochemical Purity
ROI: Region-of-interest
SBMP: Starch-Based Microparticles
SIRT: Selective Internal Radiation Therapy
SPECT: Single Photon Emission Computed Tomography
TOF: Time of Flight
VOI: Volumes-of-interests
